# Characterization of genes in guar gum biosynthesis based on quantitative RNA-sequencing in guar bean (*Cyamopsis tetragonoloba*)

**DOI:** 10.1038/s41598-019-47518-5

**Published:** 2019-07-29

**Authors:** Haiyan Hu, Haijie Wang, Yaoyuan Zhang, Baolin Kan, Yuanhao Ding, Jiaquan Huang

**Affiliations:** 1 Hainan Key Laboratory for Sustainable Utilization of Tropical Bioresource, Institute of Tropical Crops, Hainan University, Haikou, 570228 China; 2 Hainan Academy of Agricultural Sciences, Haikou, 571100 China

**Keywords:** RNA sequencing, Plant embryogenesis

## Abstract

Guar gum is an important raw material in the food, textile and oil industries, but the biosynthesis of guar gum remains unclear. To illuminate the genes involved in guar gum biosynthesis, guar beans from 30 and 40 days after flowering (DAF) were used for RNA sequencing in this study. A total of 2,535 and 2,724 preferentially expressed genes were found in 30 and 40 DAF endosperm, and 3,720 and 2,530 preferentially expressed genes were found in 30 and 40 DAF embryos, respectively. Of these, mannan synthase genes, α-galactosyltransferase genes and cellulose synthase genes were preferentially expressed in the endosperm from 30 and 40 DAF. The high expression level of these glycometabolism genes in endosperm is consistent with the expectation that the main component of guar gum is galactomannan. We believe that genes related to guar gum biosynthesis found in this study will be useful for both new variety development via genetic engineering and synthetic biology research on guar gum biosynthesis in the future.

## Introduction

Guar bean (*Cyamopsis tetragonoloba*), also known as cluster bean, is an annual herb that originates from the tropics of Asia and Africa^1^. Guar bean is a summer crop with high drought tolerance that can fix atmospheric nitrogen for plant growth, like most *Leguminosae* plants^2,3^. Guar gum is exclusively produced in the endosperm from the guar bean seeds. It accumulates during seed development around the embryo and serves as an energy resource during seed germination. Guar gum is mainly composed of galactomannan along with some moisture, protein and fibre^4^.

Guar gum has been widely used in areas including the food industry, textiles, ore flotation and oil well drilling muds. As the cheapest and healthiest natural material in the food industry, guar gum performs well in preventing ice crystals in frozen products and improving liquid-solid stability^5–7^. The soluble dietary fibre derived from hydrolysed guar gum can protect against metabolic syndrome^8,9^. Guar gum performs well in appetite control and reduces additional calorie intake, thus leading to proper weight management^10^. A novel β-mannanase gene, *RmMan5A*, recently cloned from *Rhizomucor miehei*, was expressed in *Pichia pastoris* and hydrolysed guar gum successfully^11^. The enzymatic depolymerization of guar gum does not change its core chemical structure, and thus, hydrolysed guar gum is also useful for food products^12^. In addition, hydroxypropyl guar and carboxymethyl hydroxypropyl guar as ideal, fluid-based, fracturing slurry additives are commonly used in hydraulic fracturing in shale gas extraction. In the textile industry, guar gum is used to reduce breakage of yarns and enhance the strength of fabric^1^.

Galactomannan consists of (1→4)-β-D-mannose units as backbone chains linked with (1→6)-α-D-galactose units as branch chains. Synthesis of galactomannan has been reported to be catalysed by two enzymes: mannan synthase and α-galactosyltransferase^13,14^. The guar seed β-mannan synthase (ManS) gene belongs to the cellulose synthase supergene family. ManS localizes in the Golgi and makes the β-1,4-mannan backbone of galactomannan, which was highly expressed in guar seed 25 days after flowering (DAF). Overexpressing ManS in embryogenic soybean suspension-culture cells under a seed-specific promoter resulted in higher enzyme activity in the transgenic cells^15^. 1,3-β-D-Glucan synthase purified from pea plasma needs a minor proportion of polypeptides of the total membrane protein to keep enzyme activity^16^. It has been reported that α-galactosyltransferase from *Neisseria meningitidis* could catalyse transfer of a galactosyl unit from activated UDP-Gal to glycoconjugates^17^. Serine α-galactosyltransferase is reported to be involved in protein glycosylation in plants^18^, but not in guar beans.

So far, the synthesis of guar gum in guar beans during seed development has been unclear. In this study, embryos and endosperms from 30 DAF and 40 DAF guar beans were collected for RNA sequencing. A total of 2535 and 2724 genes were identified with specific expression in endosperm from 30 DAF and 40 DAF guar bean seeds, respectively. This result implied that many genes are involved in the development of guar endosperm including the synthesis of guar gum. Gene Ontology (GO) analysis showed that the differentially expressed genes (DEGs) were obviously enriched in metabolic biological processes. Kyoto Encyclopedia of Genes and Genomes (KEGG) analysis showed that DEGs between 30 DAF and 40 DAF endosperms were enriched in metabolic pathways, especially in carbohydrate metabolism. Furthermore, one mannan synthase gene, three glycosyltransferase genes and five cellulose synthase genes were highly expressed in 30 DAF and 40 DAF endosperms, indicating that these genes may be involved in galactomannan synthesis. In addition, some storage protein and fatty acid synthesis protein-related genes were highly expressed in 30 DAF and 40 DAF embryos. These results indicated that many genes are involved in galactomannan biosynthesis in endosperm and protein and fatty acid accumulation in embryos during seed development of guar beans.

## Results

### Phenotypes of 30 DAF and 40 DAF guar beans

**Figure 1:**
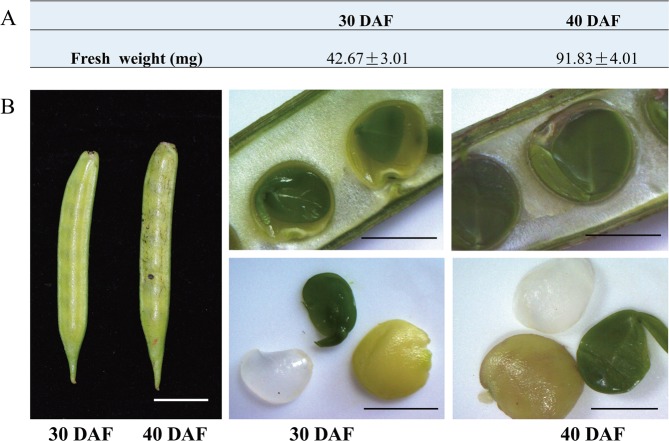
The phenotype and RNA sequencing data of guar beans. (**A)** The fresh weight of guar bean seeds from 30 and 40 DAF. (**B)** The morphology of bean pod, seed coat (pale green), embryo (dark green) and endosperm (semi-transparent white, located between the seed coat and embryo in the seed) of guar beans from 30 and 40 DAF. Scale bar: black, 0.5 cm; white, 2 cm.

A guar bean seed is composed of seed coat, embryo and endosperm. The endosperm is a white, transparent jelly surrounding the green embryo. The fresh weights of 30 and 40 DAF guar beans were 42.67 ± 3.01 mg and 91.83 ± 4.01 mg per 100 seeds, respectively (Fig. 1A), indicating a rapid substance accumulation in seeds from 30 DAF to 40 DAF. As the main weight component in seeds, endosperm was considered to undergo extreme growth during this period, which reflects the fact that 30 DAF to 40 DAF should be the most important stage for guar gum synthesis. To dissect the molecular basis of guar gum synthesis, samples from 30 DAF embryos, 40 DAF embryos, 30 DAF endosperms and 40 DAF endosperms were then collected for transcriptome sequencing (Fig. 1B).

After sequencing, raw data were first collected, and then clean reads were obtained by removing the low-quality, adaptor-polluted reads and reads with a high content of unknown base (N). For a microscale library construction method was applied for transcriptomic sequencing of samples from endosperm (reason see methods), thus more raw and clean data were obtained from samples of endosperm to ensure enough data for later use (Table 1). Due to the lack of a reference genome for guar beans, de novo assembly was applied according to the methods reported by Trinity^19^, and the unigenes were obtained by TGICL^20^. After assembly, the unique mapping ratios of the four samples ranged from 50–60% (Table 1). Moreover, the lengths of all unigenes ranged from 300 bp to over 3000 bp in the four samples, and the number of unigenes tapered off with increasing length (Fig. S1).

**Table 1:** The raw and clean data of embryo and endosperm from 30 and 40 DAF guar beans.

Sample	Raw data	Clean data	Clean Reads Ratio(%)	Total Mapped Reads	Unique Mapped Reads	Unique Mapping Ratio (%)
30DAF-Embryo	45303224	40670634	89.81	5635004	2965564	52.63
40DAF-Embryo	46940563	40484524	86.33	5315300	2703300	50.86
30DAF-Endosperm	65600392	65576694	99.96	49967156	31564364	63.17
40DAF-Endosperm	65600572	65572994	99.96	52555388	29802968	56.71

### Transcriptomic repertoires of the sequencing samples

Because there is too little total RNA in endosperm to support normal library construction, microscale transcriptomic sequencing libraries were constructed for samples from endosperm. Thus, more raw and clean reads were captured from endosperm (Table 1), and the clean reads ratios in samples from endosperm (99.96%) were much higher than those from embryo (86.33–89.81%) (Table 1). To ensure correct analysis, all reads were normalized to reduce the error between samples.

**Figure 2:**
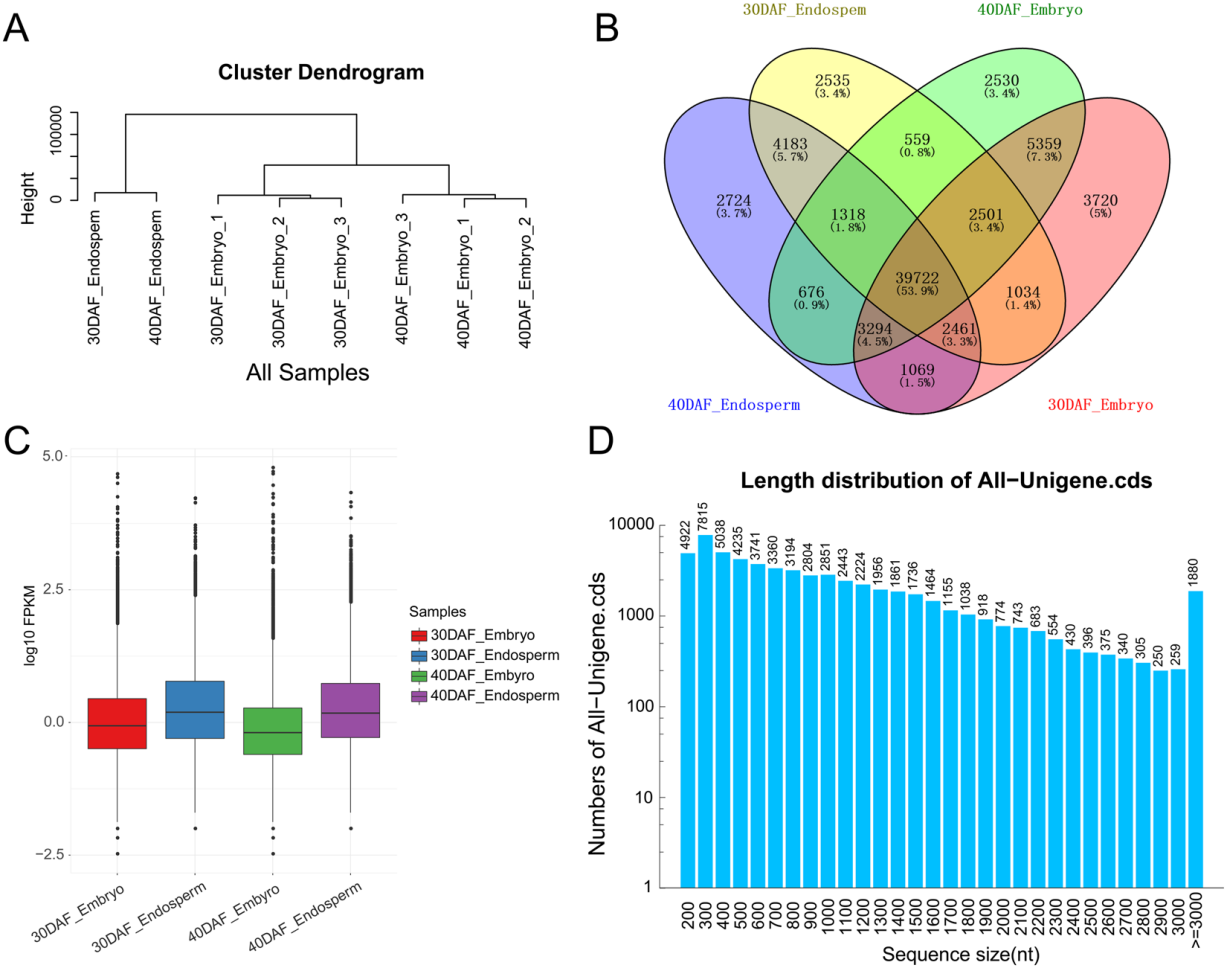
Expression analysis of guar bean seeds. (**A**) Cluster analysis of eight samples using the clean data. (**B)** Venn diagram showing the number of specific genes in 30 and 40 DAF embryo and endosperm. (**C)** The expression levels of transcripts in embryo and endosperm from 30 and 40 DAF guar beans. Transcripts from endosperm show higher expression level than the embryo. (**D)** The number and length of CDS from all unigenes.

To uncover the transcriptomic similarity between embryo and endosperm, the expression level and pattern of each transcript was used for cluster analysis. The results showed that the transcriptomes of endosperm and embryo from 30 DAF and 40 DAF clustered together independently (Fig. 2A). The results implied that the transcripts from endosperm or embryo had similar expression patterns, indicating that the gene expression differences related more to the tissue differences than to the developmental stages. Thus, we speculated that the genes and metabolic processes related to the development of embryo and endosperm were relatively independent. All expressed genes then were used for specific gene expression analysis among the samples. The results showed that most genes (39,722, 53.9%) were co-expressed in all samples. In addition, 3720 (5%) and 2530 (3.4%) genes were specifically expressed in the 30 or 40 DAF embryos, and 2535 (3.4%) and 2724 (3.7%) genes were specifically expressed in the 30 or 40 DAF endosperm, respectively (Fig. 2B). A box-plot was adopted to demonstrate the distribution of gene expression levels (Fig. 2C). The overall gene expression levels of 30 DAF embryos (median: −0.21) and 40 DAF embryos (median: −0.64) were lower than 30 DAF endosperm (median: 0.64) and 40 DAF endosperm (median: 0.58, Fig. 2C). CDS length distribution analysis showed that the number of unigenes gradually decreased with length from 200 bp to 3,000 bp and that 1880 unigenes have a CDS length over 3,000 bp, indicating that the quality of de novo assembly is high enough for further analysis (Fig. 2D).

### GO and KEGG analysis of DEGs

**Figure 3:**
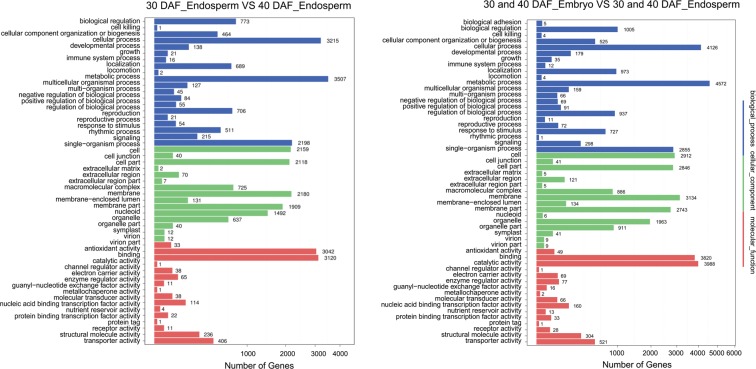
GO enrichment of DEGs from embryo and endosperm.

**Figure 4:**
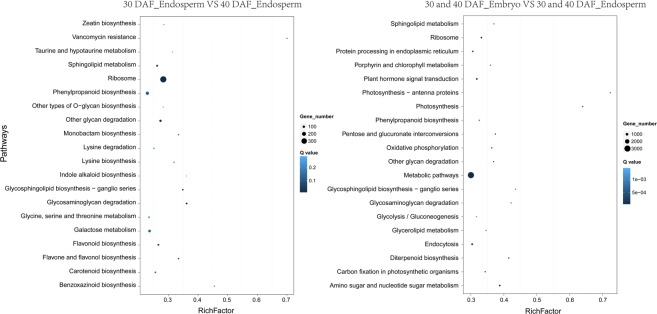
KEGG analysis of DEGs from embryo and endosperm.

DEGs between samples were first collected according to the conditions of P < 0.05 and |log2Ratios(endosperm/embryo)| > 1. Then, Gene Ontology (GO) and KEGG analyses were applied using DEGs between 30 and 40 DAF endosperm and DEGs between endosperm and embryo according to previously reported methods^21^. The annotations of the DEGs were presented using the Web Gene Ontology Annotation Plot (WEGO)^22^. The results showed an enrichment of DEGs in the cellular, metabolic and single-organism biological processes in 30 and 40 DAF endosperm (Fig. 3). In addition, pathway enrichment of DEGs between embryo and endosperm was like that of endosperm, but many more DEGs were found. KEGG analysis showed that many DEGs were involved in metabolic and ribosome pathways (Fig. 4).

### Characterization of preferentially expressed genes during guar gum synthesis

Both seed and endosperm greatly expanded from 30 to 40 DAF (Fig. 1A). Endosperm and embryo from the seed are difficult to separate before this stage because liquid jelly substances fill the seed coat at the early stage, and these two tissues can be separated at 30 to 40 DAF. We inferred that guar gum greatly accumulated during this stage. Thus, we speculated that the specific expression of genes related to guar gum biosynthesis should be obviously enriched. To characterize the genes specifically expressed in endosperm, DEGs between endosperm and embryo were first identified. To narrow the range of candidate genes, the co-expressed genes in 30 and 40 DAF endosperm and embryo were first screened. A total of 6254 downregulated and 4936 upregulated genes were found between 30 and 40 DAF endosperm and 30 and 40 DAF embryo (Fig. S2, Table S1). Then, DEGs between endosperm and embryo were identified from these co-expressed genes.

**Figure 5:**
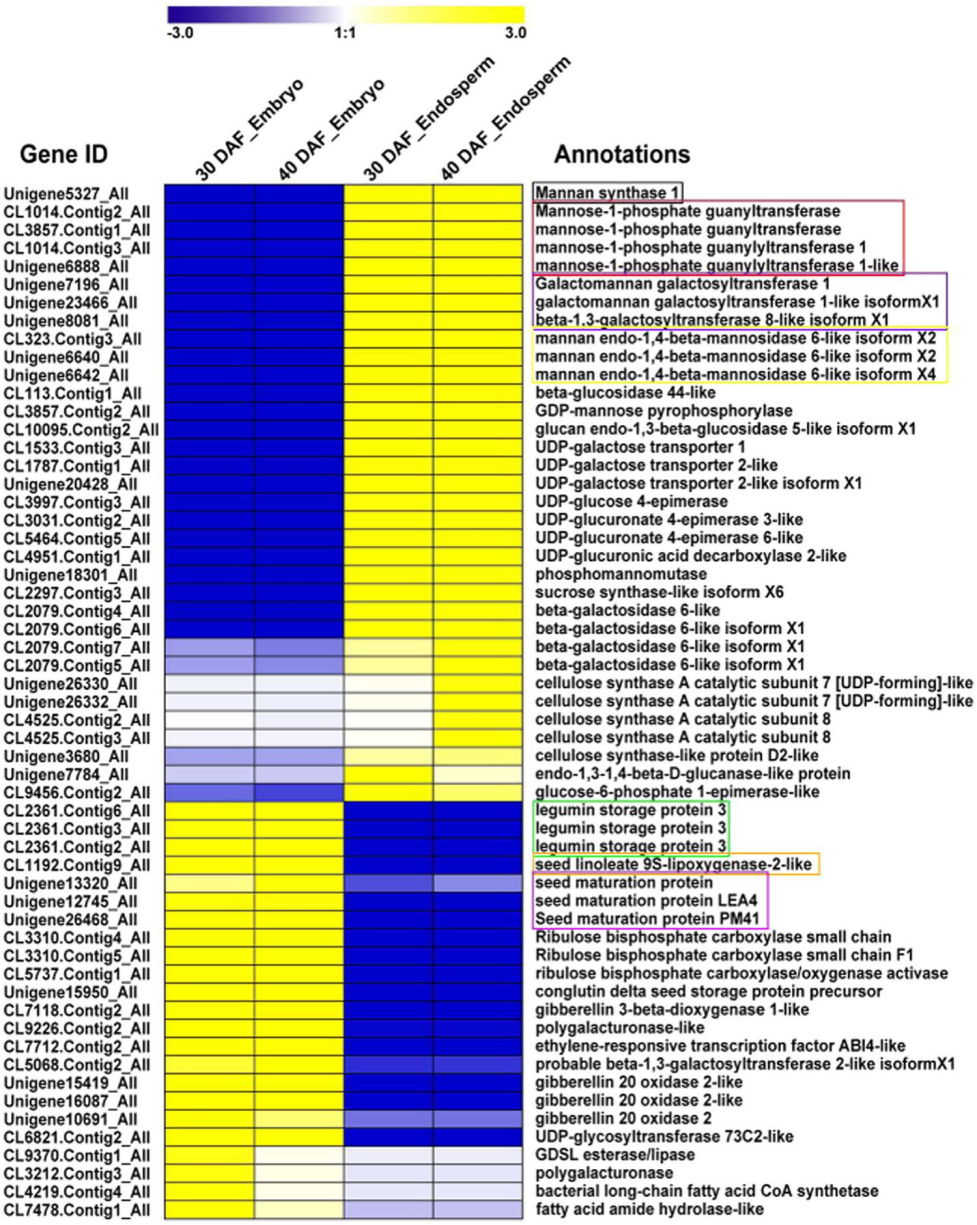
Heatmap representing DEGs between samples using the FPKM values.

To illustrate the potential genes involved in guar gum biosynthesis, carbohydrate metabolism-related genes were obtained from the candidate genes for further analysis. A heatmap was made to show expression trends of these obviously differentially expressed candidate genes between endosperm and embryo using FPKM values (Fig. 5). Interestingly, many genes were found to be specifically expressed in endosperm or embryo. In particular, one mannan synthase gene (*Unigene5327*), four mannose-1-phosphate guanyltransferase genes (*CL1014.Contig2*, *CL3857.Contig1*, *CL1014.Contig3* and *Unigene6888*), three galactosyltransferase genes (*Unigene7196*, *Unigene23466* and *Unigene8081*) and three mannan endo-1,4-beta-mannosidase 6-like genes (*CL323.Contig3*, *Unigene6640* and *Unigene6642*) were preferentially expressed in endosperm (Fig. 5). These genes were related to the biosynthesis of galactomannan, the main component of endosperm. On the other hand, three legumin storage proteins (*CL2361.Contig6*, *CL2361.Contig3* and *CL2361.Contig2*), one seed linoleate 9S-lipoxygenase (*CL1192.Contig9*) and three seed maturation proteins (*Unigene13320*, *Unigene12745* and *Unigene26468*) were highly expressed in the embryo (Fig. 5) and were thought to be related to embryo development and seed maturation.

**Figure 6:**
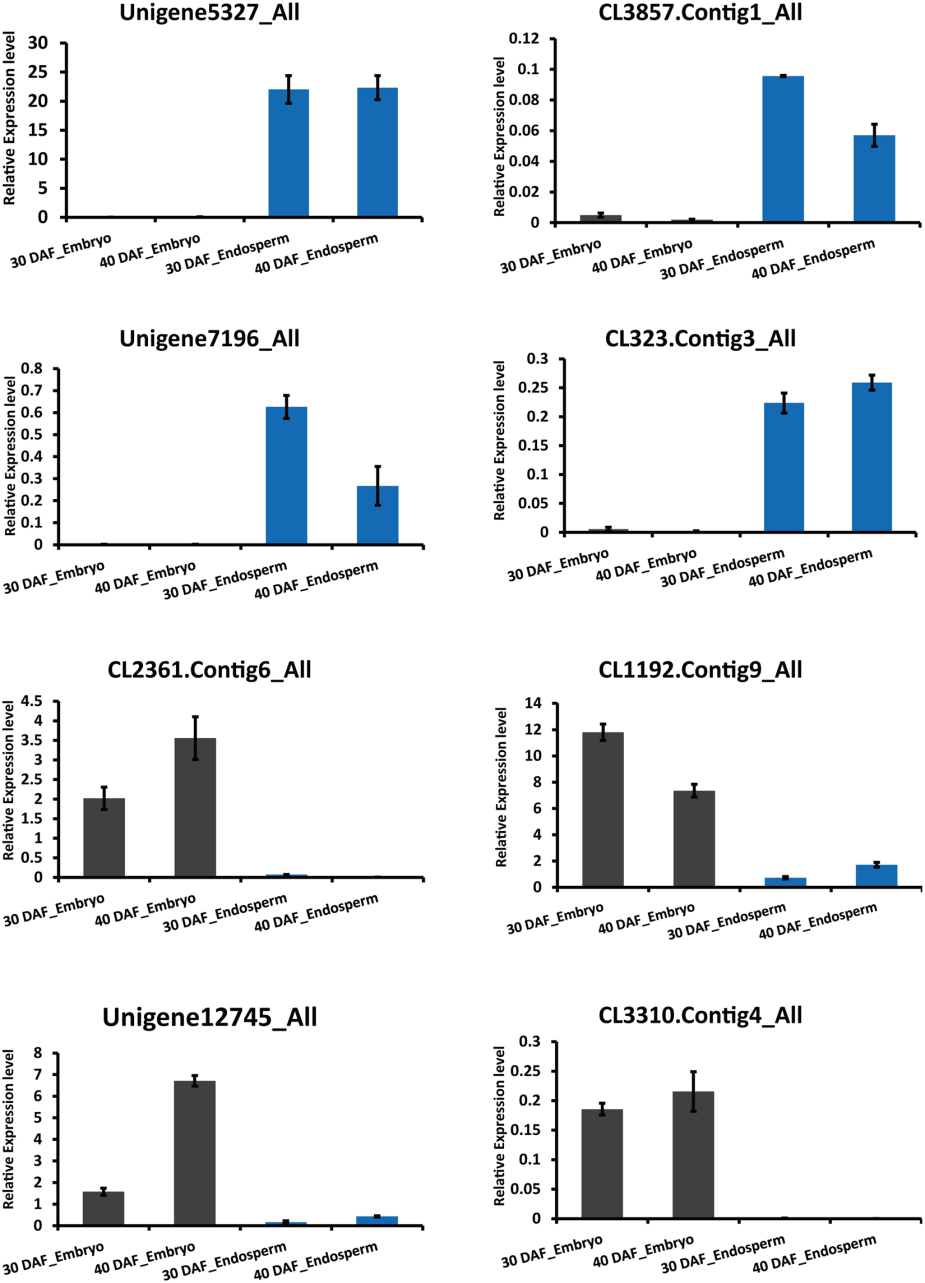
qRT-PCR validation of DEGs related to galactomannan biosynthesis or embryo development. Eight genes related to galactomannan biosynthesis or embryo development were detected by qRT-PCR. The values represent the mean ± standard deviation from 3 replicates. The tubulin encoding unigene15793 was used as an internal control.

To validate the sequencing data, eight genes peculiarly expressed in endosperm or embryo and related to galactomannan biosynthesis or embryo development were chosen for qRT-PCR (Fig. 6). The results showed that the gene expression trends were consistent with the sequencing data, indicating relatively reliable sequencing results in this study.

### Guar gum biosynthesis network in endosperm

**Figure 7:**
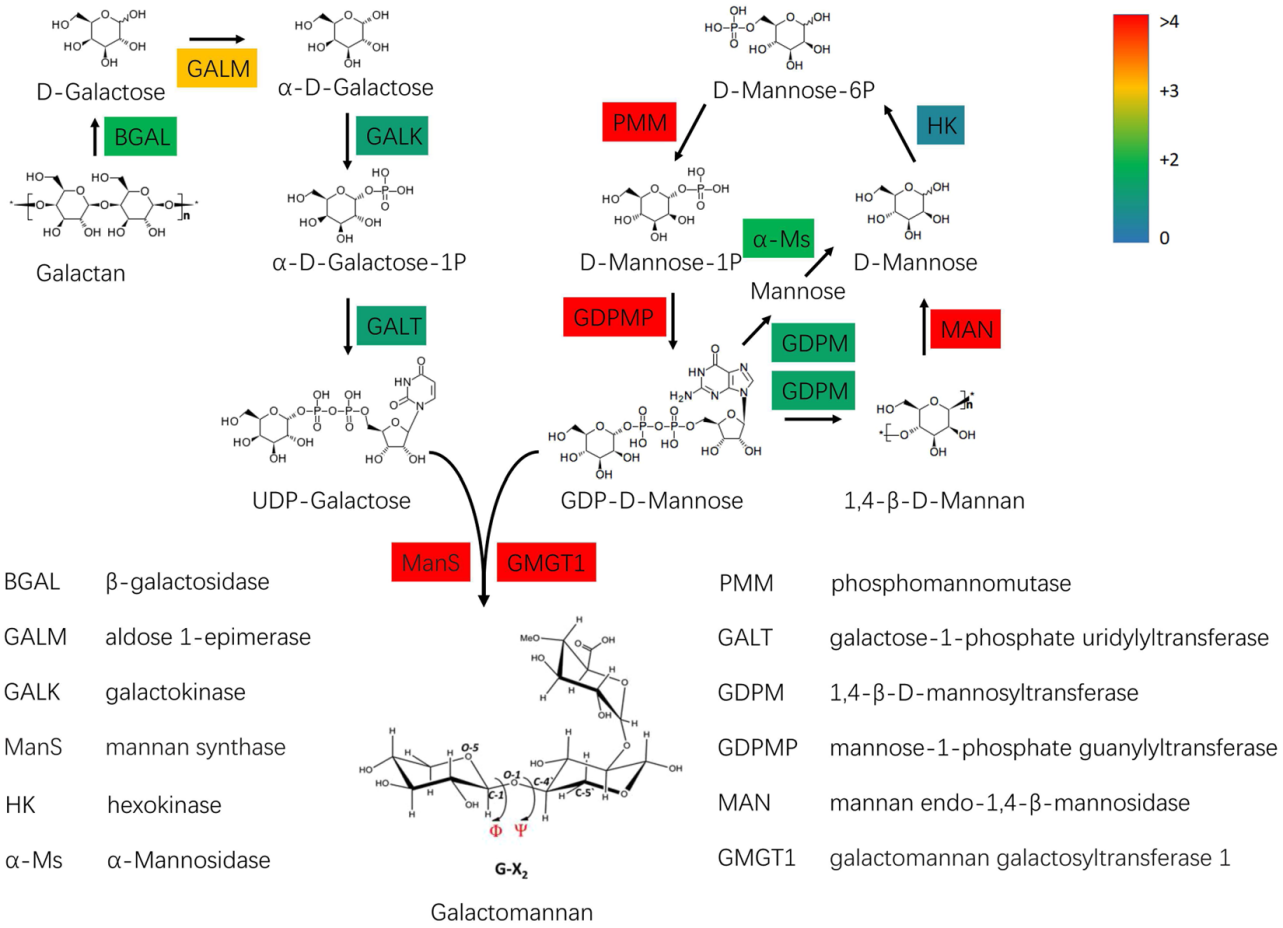
Proposed pathway of galactomannan biosynthesis in guar bean. DEGs identified by RNA-Seq were shown in chromatic blocks. Blue, green, orange and red blocks represent the expression ratios (log2(endosperm/embryo)) ranged from 0 to over 4.

According to previous studies, galactomannan is considered to be the main component of guar gum^15^. To further identify the genes involved in guar gum biosynthesis, DEGs involved in galactomannan metabolism in all samples (Table S2) were chosen for protein interaction analysis through the protein database STRING^23^. We found that most of them were upregulated (red) except for a fatty acid amide hydrolase-like protein (green). The results showed that three proteins, sucrose synthase-like protein, UDP-glucose epimerase and phosphomannomutase, were located at core positions in the network (Fig. S3). According to previous reports, mannan synthase and cellulose synthase are the enzymes necessary for guar gum biosynthesis. In our result, mannan synthase 1 was not in a core position in this network but connected with the sucrose synthase-like protein. In addition, three cellulose synthases were also found that can interact with the sucrose synthase-like protein. Thus, we propose that the sucrose synthase-like protein might play an important role in the biosynthesis of galactomannan (Fig. S3). To further illustrate the gene expression changes in guar gum biosynthesis, a metabolic pathway map of galactomannan biosynthesis in endosperm is shown in Fig. 7. The accession numbers of these genes were shown in Table S3. Nearly all genes involved in the guar biosynthesis process were found to be upregulated in endosperm relative to those in embryo. The results showed that phosphomannomutase (PMM), mannose-1-phosphate guanylyltransferase (GDPMP), mannan endo-1,4-beta-mannosidase (MAN), mannan synthase (ManS) and galactomannan galactosyltransferase 1 (GMGT1) showed significantly higher expression, an approximately 20-fold or greater increase, in endosperm. Of these, the enzymes ManS (which makes β-1, 4-linked mannan backbone) and GMGT1 (which adds galactosyl residues to the mannan backbone), the two essential enzymes for galactomannan biosynthesis, were extremely upregulated^14,24^. Gene network and pathway analysis indicated that nearly all genes involved in galactose and mannose metabolism might be involved in the biosynthesis of galactomannan.

## Discussion

Guar bean, widely cultivated in India and Pakistan, is considered to be a valuable multipurpose crop. Guar gum is obtained from the gum-containing endosperm, which comprises the outer and largely fibrous portions of the seed of guar beans. Guar gum is widely used as a raw material in many areas such as animal feeding, food processing and petroleum industries. Galactomannan, the main component of guar gum, consists of a mannose backbone with galactose side groups^25^. The galactomannan content can reach nearly 80% in guar gum, and the remaining components are moisture, protein and fibres^1^. In the past, research on guar gum mostly focused on its physicochemical properties; gene resources for guar bean were scarce, and little was known about the gum biosynthesis mechanism. Dissecting the pathways and genes involved in galactomannan biosynthesis is essential for the mechanism elucidation and genetic improvement to enhance galactomannan production, especially with the development of genome editing^26–28^, which is a cost-effective way to develop new varieties for economic plants with a small cultivation area like guar beans.

Glycosyltransferases of the cellulose synthase-like A (CslA) family were thought to catalyse β-1,4-mannan and glucomannan synthase in guar^29^. So far, only one β-mannan synthase (ManS) from guar bean has been proven functionally to be involved in β-glycan formation in plants^15^, and it was also highly expressed in the endosperm of guar in this study (Fig. 7). Because galactomannan biosynthesis requires a complex regulatory network, a series of genes must be involved in this process. Our study showed that many preferentially expressed genes in endosperm appear to be involved in the biosynthesis of galactomannan, such as *PMM*, *GDPMP*, *MAN*, *ManS* and *GMGT1*.

With the development of synthetic biology, producing the compounds of interest *in vivo* or *in vitro* by artificially designed metabolic pathways is economical and highly efficient^30^. Identification of genes and enzymes of specialized pathways is the basis of synthetic biology applications. As the functional component of guar gum, galactomannan can also be produced in many plants such as fenugreek, locust bean and tamarind. However, the ratios of mannose to galactose units range from 1:1 to 4:1 in these plants, and the genes or enzymes involved remain unclear. Moreover, the biosynthesis of mannose and galactose is common in most plant species, but not all of them can produce galactomannan, presenting an interesting scientific problem. Indeed, our study provides potentially valuable gene resources for future guar bean research, especially for galactomannan biosynthesis.

## Methods

### Plant materials

Guar beans (*Cyamopsis tetragonoloba*) used in this research were planted at Hainan University, Haikou. Guar beans from two developmental stages (30 DAF and 40 DAF) were collected. Embryo and endosperm then were separated and immediately immersed in liquid nitrogen and transferred to −80 °C storage for later use.

### RNA extraction and quantitative real-time PCR analysis

Total RNAs were extracted from frozen samples using the RNAprep pure Plant Kit (TIANGEN, DP432). Reverse-transcription reactions were performed using the PrimeScript RT reagent kit (Takara, RR047A). The Rotor-Gene Q 5plex HRM (QIAGEN) was used to detect the gene expression patterns by qRT-PCR. The Unigene15793 (encoding tubulin protein) was used as an internal control, and primers used in this research are listed in Table S4.

### Transcriptome library construction and sequencing

All RNAs collected from embryo and endosperm had rRNA removed for constructing transcriptome libraries. The mRNA was sequenced from transcriptome libraries on the Illumina HiSeq sequencing platform at BGI-Tech, Shenzhen, China. Because there is too little total RNA in endosperm to support normal library construction, a microscale library construction method was applied for transcriptomic sequencing of samples from endosperm according to reported methods^31,32^, and it was not experimentally repeated.

### De novo assembly and unigene functional annotation

Due to the lack of a reference genome, clean reads were assembled using Trinity^19^. Unigenes then were obtained after removing abundant transcripts by TGICL^20^. After assembly, unigene functional annotation was carried out with seven functional databases (NR, NT, GO, KOG, KEGG, SwissProt and InterPro). We used TransDecoder software^33^ to identity the candidate coding regions among the unigenes. For each, the longest ORF (Open Reading Frame) was selected and blasted in the SwissProt and Hmmscan databases to search for the Pfam protein homology sequences for the prediction of CDS (Coding DNA sequences).

### Identification of specifically expressed genes

Based on the assembly results, we mapped all the clean reads of each sample to the unigenes with Bowtie2 software and calculated the gene expression levels according to reported methods^34^. Base on the gene expression levels, the DEGs were identified between samples. The genes specifically expressed in endosperm were identified by a rigorous algorithm. The false discovery rate (FDR) was set at 5% to determine the P-value threshold in multiple tests and analyses by manipulating the FDR value. P < 0.001 and the absolute value of log2Ratio > 1 were used as the thresholds to judge the significance of gene expression differences. The enzymes identified in our paper were come from the DEGs clustered between embryo and endosperm (Table S1, Fig. S2). Then 31 DEGs which co-expressed between embryo and endosperm and related to galactose or mannose metabolism were screened based on the expression changes and annotations.

### GO and KEGG analysis

The unigenes that aligned to the NR database were annotated using the GO database with Blast2GO^21^. The unigenes were annotated using the KEGG database for calculating the unigene distribution of KEGG level 1 and KEGG level 2.

### Protein-protein interaction networks of DEGs

STRING software was used to analyse the protein-protein interactions of DEGs^23^. The top DEGs interactions were used to construct interaction networks.

## Supplementary information

Supplymentary Info

Dataset 1

## Data Availability

Sequence data from this article can be found in the National Center for Biotechnology Information under the following accession number: PRJNA486400.
