# Supplementary material for: Characterization of genes in guar gum biosynthesis based on quantitative RNA-sequencing in guar bean (Cyamopsis tetragonoloba)
Source: Sci Rep. 2019 Jul 29;9:10991. doi: 10.1038/s41598-019-47518-5 (PMC6662795; doi:10.1038/s41598-019-47518-5)
Supplement: Supplementary file 1 — Supplymentary Info [file 41598_2019_47518_MOESM1_ESM.pdf]

Characterization of genes in guar gum biosynthesis based on quantitative RNA-sequencing in guar bean (*Cyamopsis tetragonoloba*)

Haiyan Hu[#, 1], Haijie Wang[#, 1], Yaoyuan Zhang[1], Baolin Kan[1], Yuanhao Ding[*, 1], Jiaquan Huang[*, 1]

[#]Contributed equally; *Corresponding author

Address:

1. Hainan Key Laboratory for Sustainable Utilization of Tropical Bioresources, Institute of Tropical Agriculture and Forestry, Hainan University, Haikou, 570228, China

Corresponding author:

Jiaquan Huang

Tel: +86 0898-66260171

E-mail: jqhuang@hainu.edu.cn

This PDF file includes:

Supplementary Figs.1, 2, 3

Supplementary Tables 2, 3, 4

Supplementary Information

Figure S1: Length distribution of all unigenes.

Figure S2: Heatmap representing DEGs between endosperm and embryo using the FPKM values.

Figure S3: Protein interaction network involved in galactomannan metabolism.

Table S1: All DEGs between embryo and endosperm.

Table S2: DEGs involved in galactomannan metabolism in 30 and 40 DAF embryo and endosperm.

Table S3: The accession numbers of specific enzymes.

Table S4: List of primers used in this study.

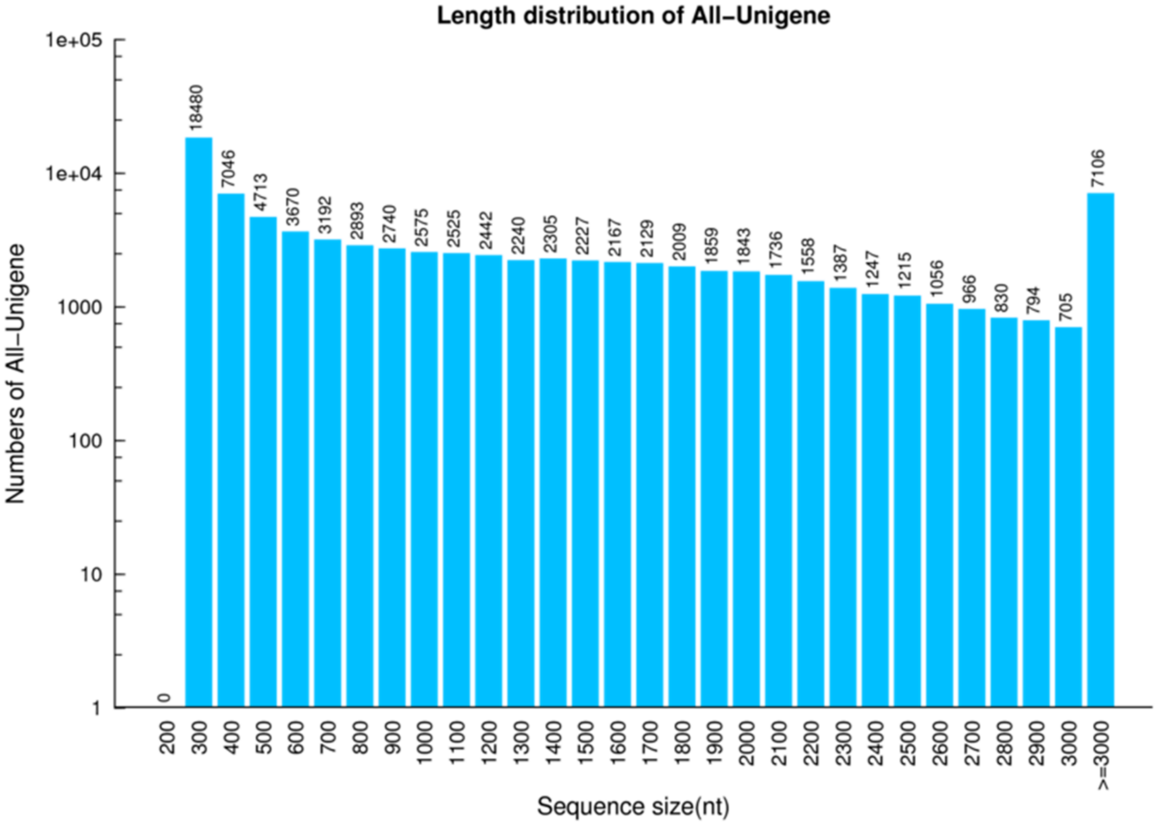

Figure S1: Length distribution of all unigenes.

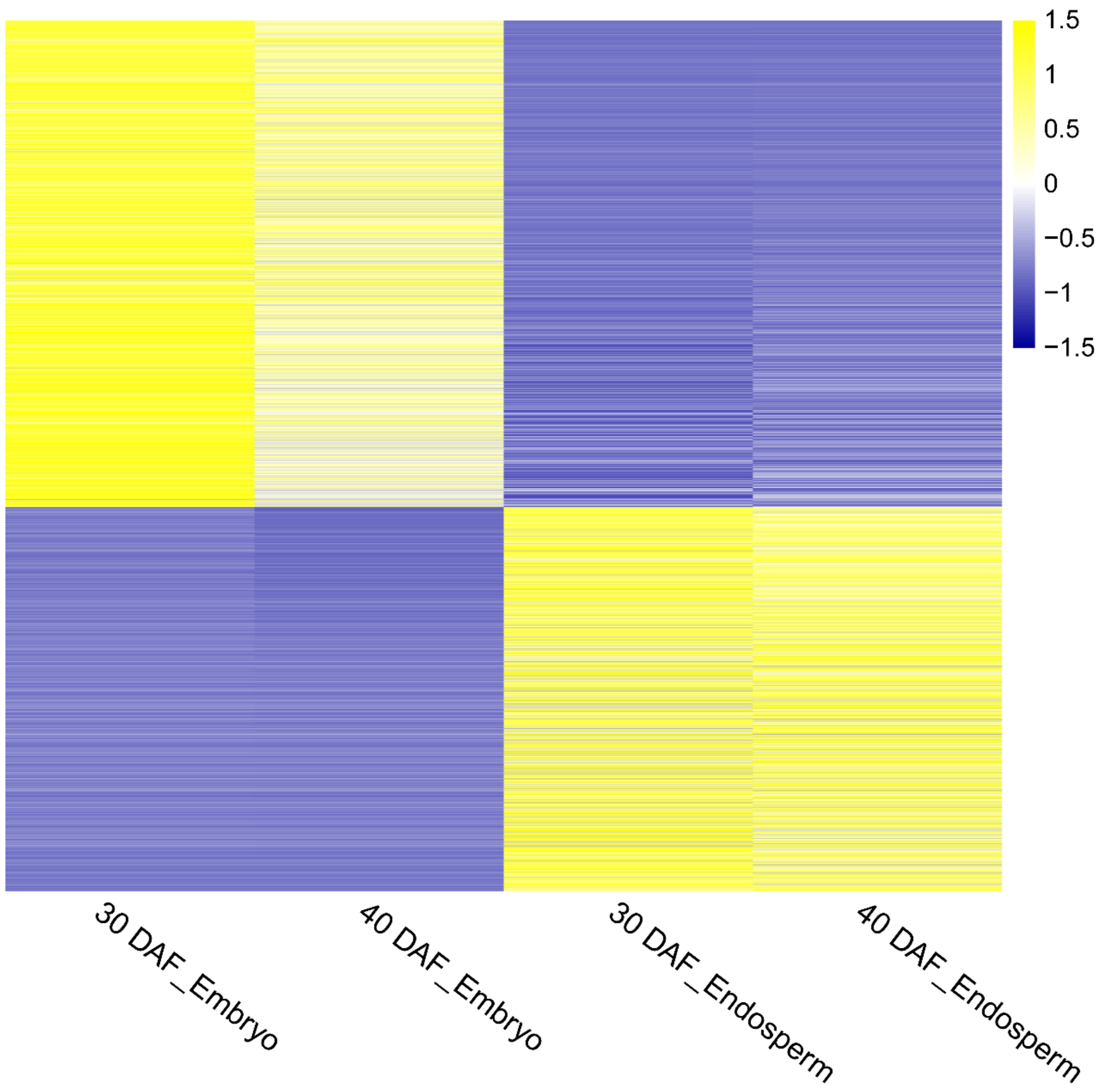

Figure S2: Heatmap representing DEGs between endosperm and embryo using the FPKM values.

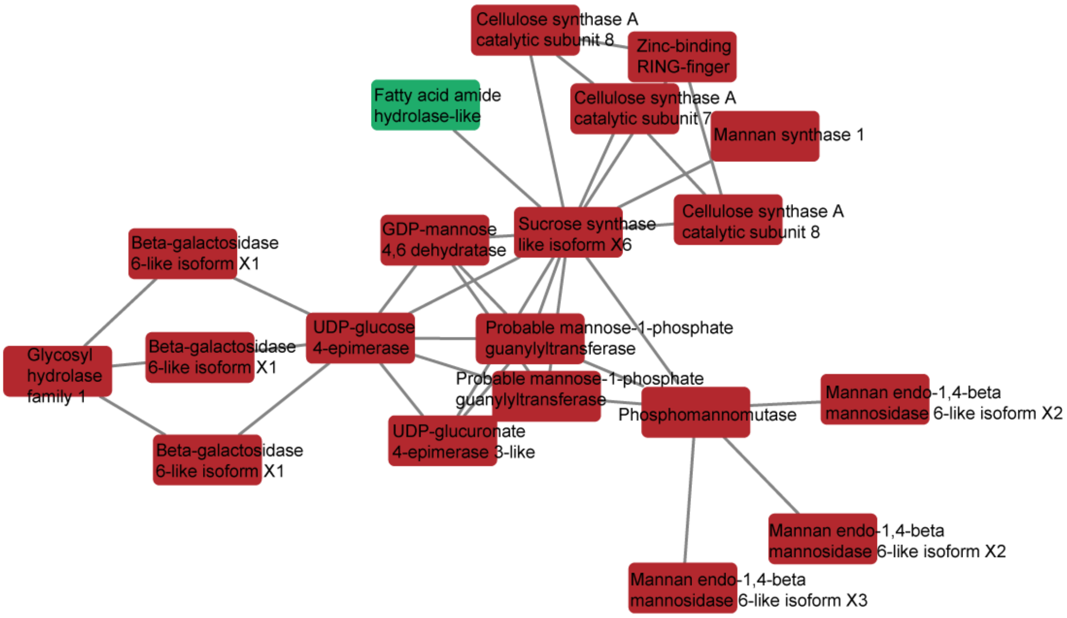

Figure S3: Protein interaction network involved in galactomannan metabolism.

Table S2: DEGs involved in galactomannan metabolism in 30 and 40 DAF embryo and endosperm.

gene_id	Embryo		Endosperm	
	30 DAF	40 DAF	30 DAF	40 DAF
Unigene5327_All	0.09	0.01	1362.61	1516.99
CL1014.Contig2_All	13.83	8.58	912.08	562.69
CL3857.Contig1_All	13.45	13.14	372.41	206.47
CL1014.Contig3_All	14.56	8.09	648.96	417.29
Unigene6888_All	12.73	14.64	593.02	317.69
Unigene7196_All	1.05	0.92	3151.71	1488.66
CL113.Contig1_All	21.55	14.89	1170.01	663.54
Unigene23466_All	1.52	1.10	576.37	436.02
CL3857.Contig2_All	11.27	12.73	319.94	169.47
Unigene4804_All	9.72	2.74	370.79	3.89
Unigene20892_All	0.75	0.29	549.34	3.46
CL10095.Contig2_All	0.81	0.92	14.65	21.95
CL1533.Contig3_All	10.89	7.80	300.49	221.24
CL1787.Contig1_All	0.81	1.30	34.44	21.82
Unigene20428_All	1.49	0.91	44.21	27.02
CL3997.Contig3_All	1.52	2.76	94.84	80.95
CL3031.Contig2_All	1.06	0.59	15.06	14.34
CL5464.Contig5_All	1.56	1.38	11.89	55.07
CL4951.Contig1_All	0.14	0.27	15.18	15.94
CL288.Contig4_All	0.34	0.20	0.26	27.48
CL323.Contig3_All	0.08	0.78	101.14	121.18
Unigene6640_All	0.17	1.41	204.62	263.92
Unigene6642_All	0.41	0.81	79.95	86.09
Unigene1765_All	0.12	0.10	0.13	21.07
Unigene8081_All	0.04	0.01	91.14	50.02
Unigene18301_All	4.48	2.32	263.65	45.50
CL2297.Contig3_All	1.58	0.31	418.70	85.13
CL2079.Contig4_All	0.01	0.01	28.19	52.00
CL2079.Contig6_All	0.14	0.13	6.97	11.85
CL2079.Contig7_All	0.42	0.05	2.75	6.08
CL2079.Contig5_All	0.39	0.12	2.67	7.68
Unigene26330_All	0.04	0.06	0.40	17.70
Unigene26332_All	0.09	0.05	0.45	16.40
CL4525.Contig2_All	0.17	0.01	0.25	15.52
CL4525.Contig3_All	0.05	0.06	0.36	13.38
Unigene3680_All	0.02	0.02	2.19	2.63
Unigene7784_All	0.16	0.12	4.55	1.37
CL9456.Contig2_All	0.46	0.01	12.83	4.00

Unigene6639_All	0.44	0.42	9.88	15.00
CL2361.Contig6_All	31376.77	47541.66	177.08	683.95
CL2361.Contig3_All	17462.65	28885.29	63.44	296.58
CL2361.Contig2_All	11439.12	16971.94	78.94	321.22
CL1192.Contig9_All	24.60	18.31	2.31	7.80
Unigene18064_All	21.43	195.45	3.99	22.60
Unigene13320_All	3.73	27.25	0.26	0.94
Unigene12745_All	191.17	1205.92	6.10	27.55
Unigene26468_All	44.71	192.97	1.32	17.97
CL3310.Contig4_All	70.63	67.82	0.01	0.01
CL3310.Contig5_All	48.83	46.93	0.01	0.01
CL5737.Contig1_All	12.59	12.06	0.01	0.01
Unigene15950_All	312.17	332.19	0.18	0.09
CL4219.Contig4_All	5.58	0.58	0.01	0.01
CL7478.Contig1_All	7.32	1.47	0.01	0.05
CL9226.Contig2_All	12.16	5.89	0.01	0.01
CL7712.Contig2_All	30.35	18.25	0.01	0.39
CL5068.Contig2_All	4.90	5.63	0.01	0.08
Unigene10691_All	6.85	3.34	0.01	0.01
Unigene15419_All	142.36	79.35	0.21	1.58
Unigene16087_All	9.88	6.23	0.01	0.01
CL6821.Contig2_All	22.41	9.11	0.26	0.09
CL9370.Contig1_All	3.02	0.49	0.01	0.01
CL3212.Contig3_All	3.18	0.65	0.03	0.03
CL7118.Contig2_All	71.17	14.62	0.71	2.50
Unigene15475_All	150.00	81.56	1.66	26.32
CL3110.Contig2_All	11.62	12.26	28.90	53.52
CL10024.Contig1_All	12.60	9.34	37.07	24.33
CL1356.Contig4_All	7.09	9.67	92.32	139.79
Unigene18220_All	9.87	4.69	12.39	15.49
CL4643.Contig1_All	12.86	4.22	46.75	2.74
CL9755.Contig1_All	3.06	2.17	19.60	21.48
CL6451.Contig1_All	287.67	108.80	7.51	6.48

Table S3:The accession numbers of these specific enzymes.

Gene name	Accession number
ManS	Unigene5327_All
GMGT1	Unigene7196_All
GALK	CL3110.Contig2_All
GALT	CL10024.Contig1_All
GALM	CL1356.Contig4_All
PMM	Unigene18301_All
HK	Unigene18220_All
GDPMP	CL1014.Contig3_All
MAN	Unigene6640_All
GDPM	CL4643.Contig1_All
α-Ms	CL9755.Contig1_All
BGAL	CL6451.Contig1_All

Table S4: List of primers used in this study.

Primers	Sequences
Unigene15793-F	GCGAGGAACAAAAGAAGCCACT
Unigene15793-R	TCGATACGCGACTTTGACCGA
Unigene5327-F	AGCTTGTGTGATAGTTCCCGAAGT
Unigene5327-R	CATGGATCTTGGGGTGGAGAC
CL3857.Contig1-F	ATGGGAATTCCAGAGGAGAGGG
CL3857.Contig1-R	CTGAGCCAAATTAGGAATCCTTTTA
Unigene7196-F	TCTCGTATTCCGGAGTCTGTGG
Unigene7196-R	CATAGCTGGACGGGTCTAAACG
CL323.Contig3-F	GAATTATGTTCCTAACCGTGCCC
CL323.Contig3-R	AATTGGCTCTGGTGGAGGACTAG
CL2361.Contig6-F	TTCGACACTGACGACCAAACTG
CL2361.Contig6-R	TTCCCATGTCGGACTGATAACAC
CL1192.Contig9-F	TGCACCCATCTAGGTCAAGGAC
CL1192.Contig9-R	GTAAACCCTTGCGTAATCCGC
Unigene12745-F	CTCACCAAACTTCGGCAGTACC
Unigene12745-R	TGCCTCCTGTATTGGTCCCA
CL3310.Contig4-F	ATGGCTTCCTCTATGCTCTCCT
CL3310.Contig4-R	GTAATGTCGTTGTTGGCCTTGC
